# The Power of Music: Enhancing Muscle Strength in Older People

**DOI:** 10.3390/healthcare7030082

**Published:** 2019-06-27

**Authors:** Nadja van den Elzen, Vera Daman, Merel Duijkers, Kim Otte, Esmée Wijnhoven, Hans Timmerman, Marcel Olde Rikkert

**Affiliations:** 1 Department of geriatrics, Radboud university medical center, 6525 GA Nijmegen, The Netherlands; 2 Donders Institute for Medical Neurosciences, Radboud university medical center, 6525 HR Nijmegen, The Netherlands; 3 Department of Anesthesiology, Pain and Palliative Medicine, Radboud university medical center, 6525 GA Nijmegen, The Netherlands

**Keywords:** elderly, muscle power, handgrip strength, music

## Abstract

Sarcopenia is a major problem occurring in the aging population. Based on previous research, music appears to have a positive influence on many aspects of life, including physical performance. This led to the question of whether listening to self-selected favorite music could improve peripheral muscle strength in older people. In this crossover study, community-dwelling people aged 65 and older were included. All participants performed handgrip strength measurements in three different circumstances: while listening to their favorite music, their most disliked music, and no music at all. As the primary outcome measurement, the within-person differences in maximum handgrip strength between the three music conditions were analyzed. A total of 153 participants (aged 73.0 ± 6 years) were included. Listening to favorite music resulted in an increase in maximum handgrip strength of +0.87 kgf (0.54–1.21, *p* < 0.001) compared to no music, and of +0.97 kgf (0.56–1.37, *p* < 0.001) compared to least favorite music. Thus, listening to favorite music has a positive effect on handgrip strength in older people. Apart from its implications for scientific grip strength measurements, this effect may be used as a fun and innocent stimulant in rehabilitation and workout classes with seniors, which could be further tested in a range of older people.

## 1. Introduction

One in three older people living at home and fifty percent of older people living in a nursing home fall at least once a year [1]. One of the causes of fall incidents is peripheral muscle dysfunction, which increases in prevalence by age [2]. Sarcopenia is present in 2 to 37 percent of community-dwelling older people, depending on the criteria [3]. In geriatric practice, frailty and muscle power are often measured using handgrip strength, which is a valid measure for peripheral muscle function [4]. Furthermore, handgrip strength is a part of frailty characteristics, predictive of mortality and sickness [5,6,7], and closely related to the amount of muscle mass in the body [8]. Improving muscle function and grip strength in older people is difficult, which warrants a search for therapeutic innovations. 

Previous research has shown positive effects of music on many aspects of life, including movement and cognition [9,10,11,12]. One study found that music therapy has beneficial psychosomatic effects and improves cognition and exercise in older people with dementia [6]. Others showed that music stimulates motivation in people who are rehabilitating after an injury [7]. In sports science, there is evidence of a positive effect of music on grip strength and performances in running and other endurance sports [13,14,15]. In addition, De Dreu et al. investigated the effects of music during exercise therapy in patients with Parkinson’s disease, who showed significant improvements in gait and gait-related activities [16]. 

As a theoretical background for these results, Bishop et al. examined the neurophysiological mechanisms associated with music in the context of physical performance [17]. They placed young athletes in an fMRI scanner to see which parts of the brain are stimulated while listening to music. They found that music with faster tempo and higher intensity stimulated structures integral to visual perception (inferior temporal gyrus), allocation of attention (cuneus, inferior parietal lobule, supramarginal gyrus), and motor control (putamen) during reactive performance. Others have shown signs of a sympathetic reaction of the nervous system to the rhythmical components of music [18,19,20]. Arousal may also be increased via extramusical associations [21]. Both stimulating and calming music are able to prepare people mentally for exercise. Stimulating music can increase the adrenergic response, increasing heartrate and breathing rate, while calming music with strong extramusical associations can conjure the right type of mental processes [21]. In addition, music might also cause a dissociation from the fatigue and discomfort that is produced by exercise [22,23]. 

A person’s preferred music—regardless of the type—may have additional qualities. For instance, the familiarity of a melody can influence its psychophysiological effects [14]. Basic research has shown that listening to a person’s favorite type of music improves functional connectivity in the default network in the brain and alters the connectivity between auditory brain areas and the hippocampus, which are involved in self-referential thought and memory encoding [24]. In a study by Bartolomei et al., favorite music had a positive effect on strength endurance in young men while performing bench press exercises. [25]. Biagini et al. also showed increased performances during an explosive exercise while listening to self-selected music [26].

This led to the hypothesis that self-selected favorite music may also have a beneficial effect on movement, peripheral muscle strength, and rehabilitation in older people. As a first test of this hypothesis, we designed a crossover trial with different types of music in healthy older people, using handgrip strength as a well-validated measure of peripheral muscle strength [4].

## 2. Materials and Methods

### 2.1. Study Population

We selected a group of healthy community dwelling people aged 65 and older, who were recruited at a senior exhibition and a senior sports class of a local physiotherapy practice. Inclusion took place in April 2018 during two randomly chosen days of the senior exhibition and two random days at the local physiotherapy practice. No exclusion criteria were set except an age below 65 years old and the inability to complete handgrip strength measurements.

All participants gave their informed consent for inclusion before they participated in the study. The study was approved by the institutional review board ‘Committee on Research involving Human Subjects Region Arnhem-Nijmegen’ with case number ‘2018-4134’ and adheres to the ethical principles as proposed by the World Medical Association Declaration of Helsinki, 7th revision (2013). The study is listed on the ISRCTN registry with study ID ISRCTN12917785. 

### 2.2. Materials and Procedure

The general characteristics of our participants were acquired with the well-validated ‘The Older Persons and Informal Caregivers Survey’ (TOPICS), to which we added questions about dominant hand and arm complaints [27]. For all participants, maximum handgrip strength was measured in three different circumstances: while listening to their favorite music, their most disliked music, and no music at all. The musical preferences were asked as an open question beforehand. Identical headphones were used to exclude a confounding effect based on different sound qualities or loudness. A handheld dynamometer (Jamar^TM^ via FysioSupplies B.V., Groningen, the Netherlands) was used to perform the measurements, following a standard protocol [18]. Handgrip strength was measured in kilogram force (kgf). 

The experiment was executed in all six possible orders of the music (Table 1). This should prevent a systematic carryover effect of tiredness and of the different types of music. Participants were divided over the six setups in the order in which they showed up at the test setting. Each participant received exactly the same instruction and had a single test session before starting the measurements. For each type of music, maximum handgrip strength was measured three times using the dominant hand. Participants listened to the music for the total duration of the handgrip strength measurements. The mean of these results was used for analysis. Inclusion, assignment of participants to the six test setups, and conduction of the study were done by four experienced medical students, all trained in grip strength measurements. 

The primary outcome measures were the within-person differences in maximum handgrip strength between the three music conditions. As a secondary outcome measure, the effect of multiple self-reported patient characteristics on handgrip strength was analyzed.

### 2.3. Statistical Analyses

To be able to show an effect size of 0.3 for the effect of favorite music versus no music with a power of 80% and a significance level of 0.05, the sample size of the study had to include at least 90 participants. The analysis of the effect of the different types of music on handgrip strength was conducted with a paired *t*-test. Cohen’s D effect sizes were calculated. A one-way ANOVA was performed to check for differences in results between the six test setups. Using an unpaired *t*-test, differences in handgrip strength in specific groups (gender, dementia, hearing problems, and arm complaints) were analyzed. To test for interactions of multiple patient characteristics with handgrip strength and with the differences in handgrip strength between the types of music, multiple linear regression and repeated measures analysis were performed, respectively. For data analysis, we used SPSS (IBM SPSS Statistics 25).

## 3. Results

**Figure 1:**
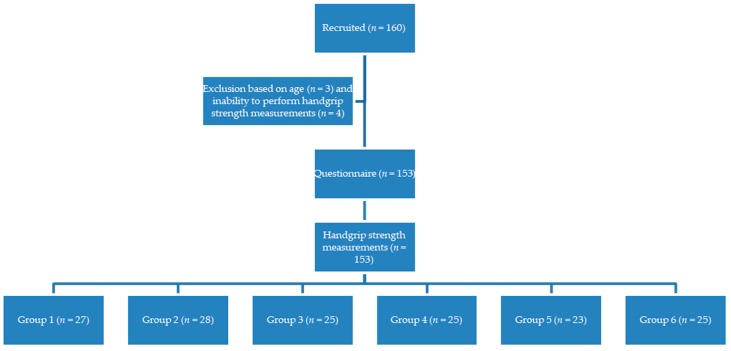
Flow chart.

One hundred and sixty people were recruited for the study, of whom 157 fitted the inclusion criteria. Three people were under 65 years of age. Due to an overwhelming enthusiasm to participate at both sites, we included more subjects than was calculated for the minimum sample size beforehand. Four participants were not able to perform the tests because of severe arm complaints, which led to a total of 153 inclusions (Figure 1). The average age was 73.0 ± 6 years old. The total number of participants in each of the six test setups slightly differed (group 1: 27 persons; group 2: 28 persons; group 3: 25 persons; group 4: 25 persons; group 5: 23 persons; group 6: 25 persons), as a consequence of the exclusions and the inclusion at two different settings. Table 2 shows the baseline information of all participants. 

No common type of favorite music could be determined. However, classical music (espescially Bach) and popular nostalgic music (especially Frank Sinatra) were mentioned most often as favorites, while Hardstyle was the least favorite type of music for many participants. 

Listening to favorite music resulted in a significant increase in maximum handgrip strength of +0.87 kgf (*p* < 0.001) compared to no music and of +0.97 kgf (*p* < 0.001) compared to least favorite music. This resulted in effect sizes of 0.38 and 0.42, respectively. There was no significant difference between the least favorite music and no music condition (Table 3). 

**Figure 2:**
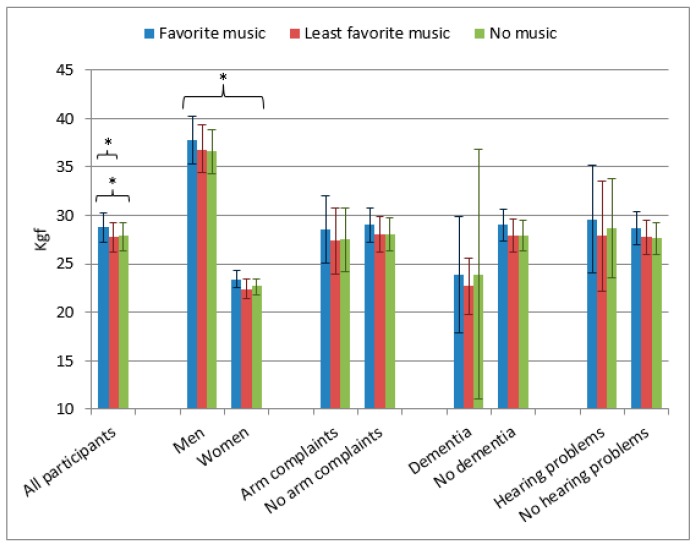
Mean handgrip strength in kgf (95% CI) related to type of music. * Significant differences (for favorite vs. least favorite music; favorite vs. no music; and men vs. women in all music categories).

The mean results of handgrip strength measurements are shown in Figure 2. There were no significant interactions between the order of presentation of the music in the six setups and handgrip strength for the different music conditions. Gender showed a significant influence on handgrip strength in all music categories (men achieved better results than women). Dementia, hearing problems, and arm complaints did not result in significant differences in maximum handgrip strength. 

Multiple linear regression analysis showed three factors with a significant correlation to handgrip strength: gender (male participants showed better results, *p* <0.001), mobility (worse mobility gave worse handgrip strength, *p* = 0.024), and weight (the heavier the better, *p* = 0.002). When using the repeated measures function, gender showed a significant effect on the music related difference in handgrip strength (*p* = 0.001). This effect was mainly caused by a 1.15 kgf (±2.19) grip strength difference in men when listening to their favorite music compared to no music, in comparison to a grip strength difference in women of 0.71 Kgf (±2.03). 

## 4. Discussion

The aim of this study was to investigate the effect of self-selected favorite music on peripheral muscle strength and exercise in older people, thereby testing a possible new and fun way to stimulate them while training. We found that older people show a significant increase in maximum handgrip strength while listening to their favorite music when compared to their least favorite music or no music at all. To our knowledge, this is the first time the effect of favorite music on handgrip strength in older people was investigated. 

Previous studies have shown the influence of music on different aspects of life, like movement, cognition, and psychological functions [9,10,11,12,13,14,15,16,17,18,19,20,21,22,23,24,25,26]. Stimulating music has been used in multiple trials regarding physical performance with generally positive results [13,14,19]. The use of self-selected favorite music during exercise has also shown positive effects [15,25,26]. These results are in line with our findings. In addition, we found a significant correlation between handgrip strength and gender, mobility, and weight, which is consistent with findings in previous studies [28,29,30].

There are, however, some limitations to our study. Firstly, most of our participants were living at home, and many of them were walking independently, which limits the external validity for more frail or institutionalized older persons. Future research might therefore also aim for more vulnerable groups. Secondly, the effect of background music at the senior exhibition may have influenced results. We tried to prevent this as much as possible by using over-ear headphones to exclude ambient noise. Thirdly, participants were able to freely choose their own favorite and least favorite music. Some of them, however, had no real preference or were not able to name a song by themselves, in which case the researchers named artists and songs that were widely used by other participants to inspire them. In this case, the effect of (least) favorite music may have been distorted. Furthermore, participants and researchers were not blinded for the different types of music during measurement of handgrip strength. We tried to keep any influence by the researchers to a minimum by giving each participant exactly the same explanation during the strictly protocolized tests. 

## 5. Conclusions

This study shows that listening to self-selected favorite music has a positive effect on handgrip strength in older people. Though the absolute difference in strength gained seems modest, for a frail person, one kgf may make the difference in being able or not able to stand up. Apart from its implications for scientific grip strength measurements, this effect may be used as a fun and innocent stimulant in rehabilitation and workout classes with seniors, which could be further tested in a range of older people. 

## Figures and Tables

**Table 1:** Order of grip strength measurement conditions.

**1.**	Test ^1^	Favorite music	Least favorite music	No music
**2.**	Test	Least favorite music	Favorite music	No music
**3.**	Test	Favorite music	No music	Least favorite music
**4.**	Test	Least favorite music	No music	Favorite music
**5.**	Test	No music	Favorite music	Least favorite music
**6.**	Test	No music	Least favorite music	Favorite music

^1^ Note: Each participant received the instructions and had a single test session.

**Table 2:** Patient Characteristics.

Descriptive	Number	Percentage
Total number of participants included	153	100%
Male	57	37.3%
Living situation Independently, alone Independently, with others Institutionalized	42 110 1	27.5% 71.9% 0.7%
Mobility: walking problems No Mild Moderate Severe	88 44 12 4	57.5% 28.8% 7.8% 2.6%
One or more falls in the last 12 months	25	16.3%
Dementia	3	2.0%
Hearing problems	22	14.4%
Receiving home care	10	6.5%
Dominant hand		
Right	128	83.7%
Arm complaints	49	32.0%

Note: all health problems were self-reported.

**Table 3:** Mean difference in handgrip strength related to the type of music.

Compared Music Conditions	Mean Difference in Handgrip Strength in kgf (95% CI)	*p*-Value	Cohen’s D
Favorite music vs. no music	+0.87 (0.54–1.21)	<0.001	0.38
Favorite music vs. least favorite music	+0.97 (0.56–1.37)	<0.001	0.42
Least favorite music vs. no music	−0.09 (−0.49–0.31)	0.191	-
